# Neuropilin-1 inhibition suppresses nerve growth factor signaling and nociception in pain models

**DOI:** 10.1172/JCI183873

**Published:** 2024-11-26

**Authors:** Chloe J. Peach, Raquel Tonello, Elisa Damo, Kimberly Gomez, Aida Calderon-Rivera, Renato Bruni, Harsh Bansia, Laura Maile, Ana-Maria Manu, Hyunggu Hahn, Alex R.B. Thomsen, Brian L. Schmidt, Steve Davidson, Amedee des Georges, Rajesh Khanna, Nigel W. Bunnett

**Affiliations:** 1 Department of Molecular Pathobiology, College of Dentistry and; 2 Pain Research Center, New York University, New York, New York, USA.; 3 Department of Anesthesiology, College of Medicine, University of Cincinnati, Cincinnati, Ohio, USA.; 4 Translational Research Center, College of Dentistry,; 5 Department of Oral and Maxillofacial Surgery, College of Dentistry, and; 6 Department of Neuroscience and Physiology, Neuroscience Institute, Grossman School of Medicine, New York University, New York, New York, USA.

**Keywords:** Cell biology, Neuroscience, Pain, Signal transduction

## Abstract

Nerve growth factor (NGF) monoclonal antibodies inhibit chronic pain, yet failed to gain approval due to worsened joint damage in osteoarthritis patients. We report that neuropilin-1 (NRP1) is a coreceptor for NGF and tropomyosin-related kinase A (TrkA) pain signaling. NRP1 was coexpressed with TrkA in human and mouse nociceptors. NRP1 inhibitors suppressed NGF-stimulated excitation of human and mouse nociceptors and NGF-evoked nociception in mice. NRP1 knockdown inhibited NGF/TrkA signaling, whereas NRP1 overexpression enhanced signaling. NGF bound NRP1 with high affinity and interacted with and chaperoned TrkA from the biosynthetic pathway to the plasma membrane and endosomes, enhancing TrkA signaling. Molecular modeling suggested that the C-terminal R/KXXR/K NGF motif interacts with the extracellular “b” NRP1 domain within a plasma membrane NGF/TrkA/NRP1 of 2:2:2 stoichiometry. G α interacting protein C-terminus 1 (GIPC1), which scaffolds NRP1 and TrkA to myosin VI, colocalized in nociceptors with NRP1/TrkA. GIPC1 knockdown abrogated NGF-evoked excitation of nociceptors and pain-like behavior. Thus, NRP1 is a nociceptor-enriched coreceptor that facilitates NGF/TrkA pain signaling. NRP binds NGF and chaperones TrkA to the plasma membrane and signaling endosomes via the GIPC1 adaptor. NRP1 and GIPC1 antagonism in nociceptors offers a long-awaited nonopioid alternative to systemic antibody NGF sequestration for the treatment of chronic pain.

## Abstract 2

Neuropilin-1 and G Alpha Interacting Protein C-terminus 1 are necessary for nerve growth factor-evoked pain and are non-opioid therapeutic targets for chronic pain.

## Introduction

Nerve growth factor (NGF) was identified by its ability to stimulate growth of sympathetic neurons (1). Tropomyosin-related kinase A (TrkA), a receptor tyrosine kinase (RTK), mediates the neurotrophic actions of NGF (2). After NGF binds to TrkA at peripheral nerve terminals, NGF/TrkA signalosomes are retrogradely transported to the soma, where they regulate transcription (3, 4). p75^NTR^, a receptor for NGF and pro-NGF (5, 6), activates proapoptotic signaling pathways (7). NGF and TrkA also mediate pain (8). Although NGF and its receptors have been intensively studied in the context of neuronal development and pain, inadequate understanding of NGF signaling has hampered the approval of NGF-directed therapeutics.

Chronic pain afflicts twenty percent of the population, yet is inadequately treated by nonsteroidal antiinflammatory drugs and opioids, which lack efficacy and have life-threatening side effects. NGF/TrkA is one of the few nonopioid targets for chronic pain validated in patients. NGF secreted by injured and diseased tissues activates TrkA on nociceptors to evoke sensitization and expression of neuropeptides, receptors, and ion channels that mediate pain (8). NGF-stimulated neuronal sprouting may also contribute to pain (8). NGF and TrkA have been implicated in pain associated with inflammation, nerve injury, and cancer (8). The central role of TrkA and NGF in pain is evident in patients with hereditary sensory and autonomic neuropathy (HSAN) types IV and V, where pathological insensitivity to pain results from loss-of-function mutations in TrkA (9) and NGF (10), respectively. Although mAbs are analgesic in osteoarthritic patients (11), they failed to gain FDA approval due to worsening joint damage in some individuals (12). The identification of nociceptor-enriched mediators of NGF-induced pain may facilitate development analgesics devoid of the adverse effects of systemic NGF sequestration with mAbs.

Neuropilin-1 (NRP1) is a type I transmembrane protein discovered for its role in axon guidance (13). Transcriptome analyses confirms that NRP1 is conserved between rodent and human nociceptors (14). Lacking a catalytic domain, NRP1 does not transduce signals, but rather acts as a coreceptor for unrelated families of proteins, including VEGF-A (15). NRP1 is a coreceptor with VEGF receptor 2 (VEGFR2) that enhances VEGF-A–induced angiogenesis. NRP1 overexpression in cancers spurred the development of vesencumab/MNRP1685A mAb to inhibit tumorigenesis (16). NRP1 is also implicated in VEGF-A–mediated pain (17, 18). Since NGF possesses a prospective NRP1-binding motif, we investigated the hypothesis that NRP1 is a coreceptor for NGF/TrkA-evoked pain.

## Results

### NRP1 binds human β-NGF.

**Figure 1:**
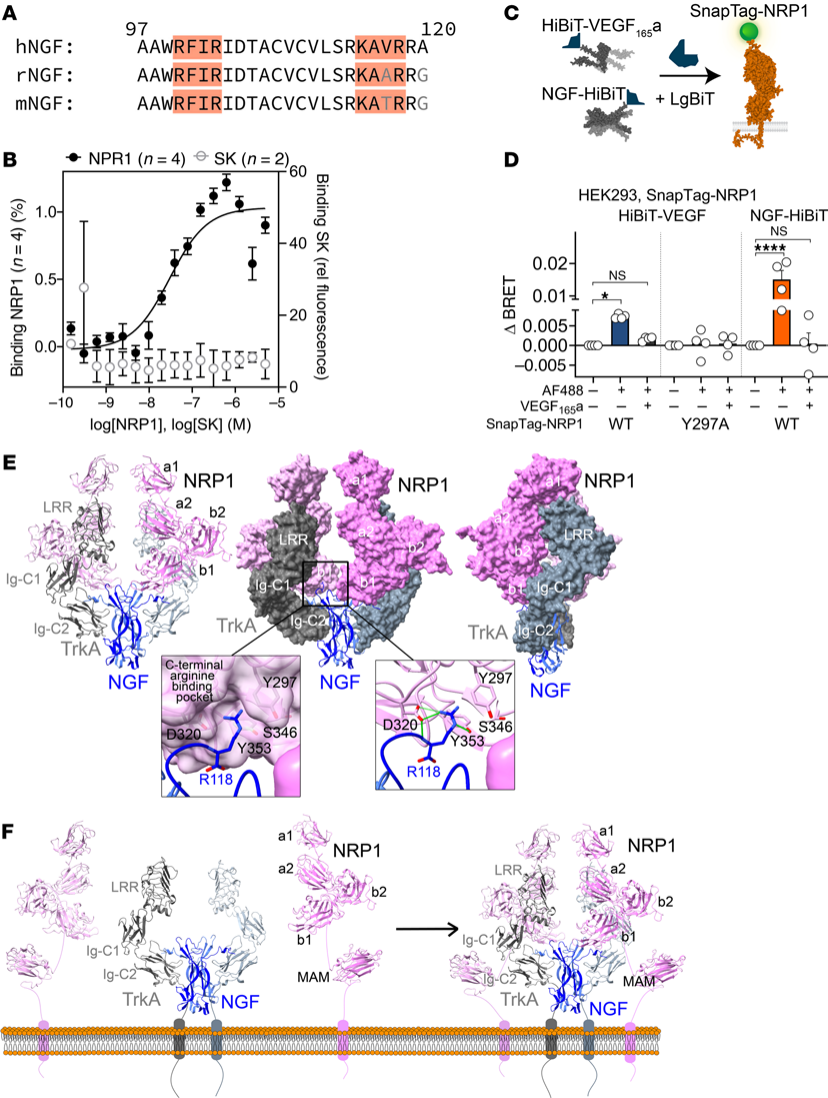
NGF/NRP1/TrkA interactions. (**A**) CendR motifs (highlighted) of NGF C-terminus numbered according to mature βNGF (1–120 equivalent to proNGF 122–241). h, Homo sapiens; r, Rattus norvegicus; m, Mus musculus. (**B**) MST interaction assay between fluorescent human βNGF and NRP1 (residues 22–644) or staphylokinase (SK, negative control). *n* = 2 or 4 independent experiments. Data are represented as mean ± SEM. (**C** and **D**) BRET assay of VEGF or NGF proximity to NRP1 in HEK293T cells. Supernatant from cells secreting HiBiT-tagged VEGF_165_a or NGF was reconstituted with recombinant LgBiT and furimazine, forming the bioluminescent donor. HEK293T cells expressing WT SnapTag-NRP1 or VEGF_165_a binding-dead mutant (Y297A) were labeled with SNAPTag-Alexa Fluor 488 (AF488) and incubated with supernatant. BRET was measured between HiBiT-VEGF_165_a and SnapTag-NRP1 (WT or Y297A) or HiBiT-tagged NGF and SnapTag-NRP1 (WT). Cells were preincubated with vehicle or unlabeled VEGF_165_a followed by luminescent growth factor. BRET was compared with negative control (HiBiT/LgBiT only lacking AF488). *n* = 4 independent experiments. Data are represented as mean ± SEM. *P < 0.05; ****P < 0.0001, 1-way ANOVA, Šídák’s multiple comparisons. (**E** and **F**) Ternary complex of human NGF/TrkA/NRP1 generated using constraint-driven computational docking. Cartoon and surface representation of TrkA (gray), NGF (blue), and NRP1 (pink) are shown. (**E**) NGF/TrkA/NRP1 model and conserved interactions at the NGF/NRP1 interface suggest a 2:2:2 stoichiometry with 1 NRP1 molecule interacting with 1 TrkA molecule and the NGF dimer. Views of the NRP1/TrkA complex in surface representation represent complementarity between NRP1 and TrkA. The inset shows binding of NGF C-terminal R118 (blue) to conserved residues (pink, Y297, D320, S346, Y353) in C-terminal arginine-binding pocket of the NRP1 b1 domain (predicted hydrogen bonds in green). (**F**) Proposed cell surface NGF/TrkA/NRP1 complex. Membrane proximal MAM NRP1 domains are included to propose a sterically feasible membrane-tethered NGF/TrkA/NRP1 complex. Membrane linkers and transmembrane regions are not derived from structures and are not to scale.

NRP1 is a coreceptor for diverse proteins, including neuronal guidance molecules (semaphorins) (19), growth factors (VEGF-A) (15), cell-penetrating peptides (20), and viruses (SARS-CoV-2) (13). NRP1 ligands share a C-terminal basic motif (C-end rule, “CendR” motif, R/KXXR/K), which interacts with extracellular “b” domains of NRP1 (15, 19). Inspection of the amino acid sequence of mature NGF identified 2 C-terminal CendR R/KXXR/K motifs that are conserved between rodents and humans (Figure 1A).

Microscale thermophoresis (MST) was used to analyze interactions between fluorescent human NGF and the extracellular a1a2b1b2 domain of human NRP1. NGF interacted with NRP1 with nanomolar affinity (*K_d_* = 35.5 ± 4.7 nM, *n* = 4, Figure 1B). No interaction was detected between fluorescent NGF and staphylokinase (negative control). The measured NGF/NRP1 affinity is lower than the reported affinities of NGF for TrkA and p75^NTR^ (1–15 nM; refs. 21, 22), but is comparable to affinity of VEGF_165_a for NRP1 (*K_d_* 9–120 nM; refs. 23, 24).

NGF binding to NRP1 in cells was analyzed using bioluminescence resonance energy transfer (BRET) (Figure 1C). VEGF_165_a (positive control known to interact with NRP1) or NGF was genetically fused to an 11 amino acid fragment (HiBiT) of nanoluciferase (NanoLuc). HiBiT has a high affinity for the complementary LgBiT fragment. HiBiT-tagged growth factors were expressed in HEK293T cells, secreted into supernatant, and conjugated to recombinant LgBiT to form full-length and catalytically active NanoLuc. Upon addition of furimazine substrate, complemented NanoLuc emits bioluminescence. Supernatant containing HiBiT-tagged growth factor was incubated with cells expressing SnapTag–NRP1 labeled with SNAP–Surface Alexa Fluor 488 fluorophore (SnapTag-AF488, +) or lacking fluorophore (–). If proteins are in close proximity (<10 nm), luminescent growth factor can act as a BRET donor to fluorescent NRP1. BRET was detected between luminescent VEGF_165_a and SnapTag-NRP1 (Figure 1D). Preincubation of cells with an excess of VEGF_165_a (10 nM) abolished the BRET signal. No BRET signal was detected when luminescent VEGF_165_a was incubated with cells expressing a binding-dead NRP1 mutant (Y297A) in the b1 domain (25, 26). Importantly, BRET was detected between NGF-HiBiT and SnapTag-NRP1, and preincubation with unlabeled VEGF_165_a inhibited this response. The results from analysis of interactions between recombinant proteins and ligand-binding studies in intact cells provide evidence that NGF directly binds NRP1 with nanomolar affinity.

### Modeling an NGF/TrkA/NRP1 ternary complex.

Ternary complexes of human NGF/TrkA/NRP1 were generated through an information-driven computational docking protocol using HADDOCK 2.4 (27). The complexes were analyzed against available biochemical data. Binding interactions of sterically feasible complexes in the cellular context were then analyzed at the molecular level. NRP1 is composed of 5 extracellular domains (ECDs) from N- to C-terminus – a1, a2, b1, b2 and meprin, A-5 protein, and receptor protein-tyrosine phosphatase mu (c/MAM) (28). The TrkA ECD comprises an N-terminal leucine-rich repeat (LRR) domain and C-terminal immunoglobulin domains (Ig-C1, Ig-C2) (29). The crystal structure of NGF/TrkA complex reveals a 2:2 stoichiometry (PDB 2IFG). Our docked model of NGF/TrkA/NRP1 suggests a 2:2:2 stoichiometry with 1 NRP1 molecule interacting with 1 TrkA molecule and the NGF dimer (Figure 1E and Supplemental Table 1; supplemental material available online with this article; https://doi.org/10.1172/JCI183873DS1). The model suggests that the NRP1 b1 domain predominantly interacts with the NGF C-terminus while also interfacing with an interdomain region between Ig-C1 and Ig-C2 domains of TrkA. In this model, the NRP1 a1 and a2 domains interface with the top of the TrkA LRR domain and the NRP1 b2 domain slots in the curved space between TrkA Ig-C1 and LRR domains contributing to shape complementarity between TrkA and NRP1 (Figure 1E). NGF has 2 C-terminal CendR R/KXXR/K motifs that mediate interaction of other growth factors with NRP1 b1 domains (20, 30, 31). In our model, R118 from CendR motif K_115_AVR_118_ binds to C-terminal arginine binding pocket of the NRP1 b1 domain through hydrogen bonding interactions between NGF R118 and NRP1 conserved residues Y297, D320, and Y353 (Figure 1E inset). Other NRP ligands also possess a C-terminal arginine that binds to a C-terminal arginine binding pocket of the NRP1 b1 domain through similar molecular interactions (31, 32). The membrane proximal MAM domain of NRP1, extending from the NRP1 b2 domains, is proposed to orient the other ECDs of NRP1 away from the membrane for protein/protein interaction (28). In the NGF/TrkA/NRP1 docked model, the NRP1 b2 domain lies perpendicular to the NGF/TrkA 2-fold symmetry axis with solvent accessible C-termini pointing toward the membrane (Figure 1E). This orientation suggests that it is possible to extend the MAM domain from the b2 domain toward the membrane while NRP1 is still bound to the NGF/TrkA complex, resulting in a sterically permissible membrane tethered NGF/TrkA/NRP1 complex (Figure 1F). TrkA Asn-linked glycosylation can regulate its localization and activity (33). Although nonstandard residues were excluded from docking calculations, the NGF/TrkA/NRP1 docked model shows that NRP1 binding to TrkA does not clash with Asn-linked glycosylations on TrkA, suggesting sterically feasible binding of NRP1 to glycosylated TrkA in a cellular context (Supplemental Figure 1). Thus, our analysis provides structural insights into the NRP1 coreceptor function for NGF/TrkA signaling analogous to a recent structural study of NRP1 coreceptor function with an unrelated family of proteins (34).

### TrkA and NRP1 are coexpressed in mouse and human DRG neurons.

**Figure 2:**
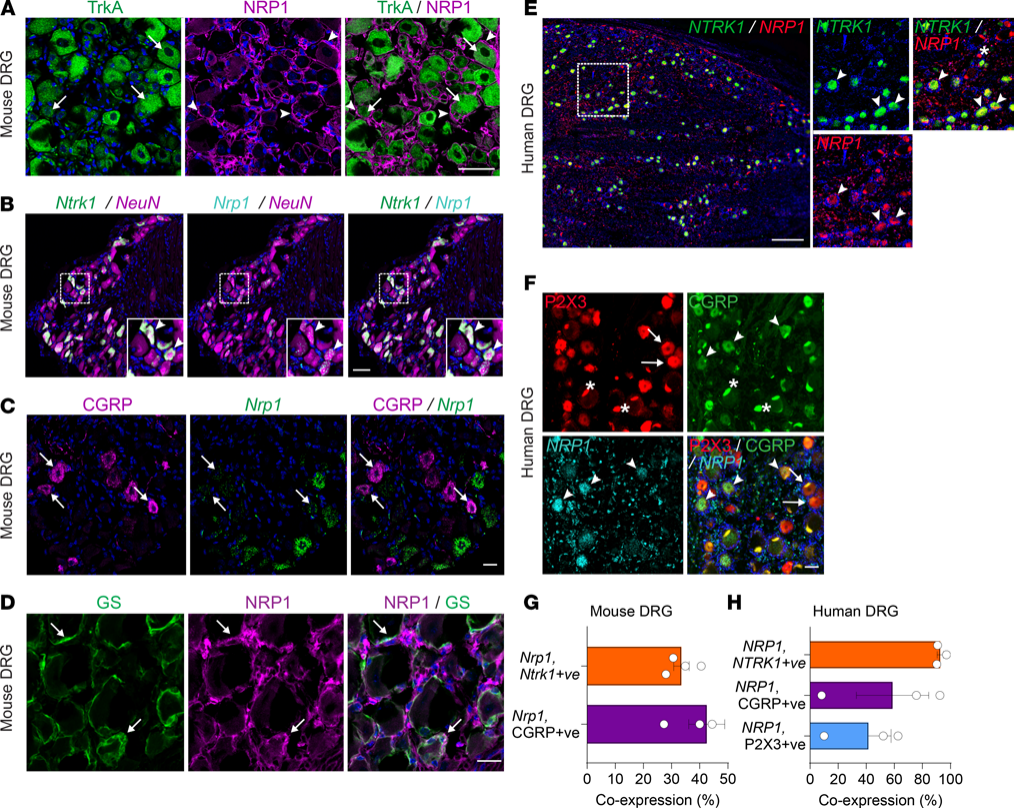
TrkA and NRP1 are coexpressed in DRG. (**A**) Immunofluorescence detection of TrkA and NRP1 in mouse DRG. TrkA was largely intracellular (arrows), whereas NRP1 was localized to the plasma membrane (arrowheads). Scale bar: 50 μm. (**B**) RNAScope detection of *Ntrk1* (TrkA) and *Nrp1* (NRP1) mRNA in mouse DRG neurons identified by NeuN immunofluorescence. Arrowheads indicate neurons coexpressing *Ntrk1* and *Nrp1*. Scale bar: 50 μm. (**C**) Immunofluorescence detection of CGRP and RNAScope detection of *Nrp1* mRNA in mouse DRG. Arrows indicate neurons coexpressing CGRP and *Nrp1*. Scale bar: 20 μm. (**D**) Immunofluorescence detection of NRP1 and GS in mouse DRG. Arrows indicate satellite glial cells expressing NRP1. Scale bar: 50 μm. (**E**) RNAScope detection of *NTRK1* and *NRP1* mRNA in human DRG. Arrowheads indicate neurons coexpressing *NTRK1* and *NRP1*. Scale bar: 500 μm. (**F**) Immunofluorescence of P2X3 and CGRP and RNAScope detection of *NRP1* mRNA in human DRG. Arrowheads indicate neurons coexpressing CGRP and *NRP1*. Arrows indicate neurons expressing P2X3 but not *NRP1*. Scale bar: 50 μm. *Denotes fluorescence in human neurons due to lipofuscin. Nuclei shown in blue. (**G**) Percentage of mouse DRG neurons expressing *Ntrk1* or CGRP that coexpress *Nrp1*. (**H**) Percentage of human DRG neurons expressing *NTRK1*, CGRP, or P2X3 that coexpress *NRP1*. **A**–**F** show representative images from *n* = 4–5 mice and *n* = 3 humans. **G** and **H** show hybridized positive neurons (%) from *n* = 3–4 mice and *n* = 3 humans.

To determine whether NRP1 could function as an NGF/TrkA coreceptor in nociceptors, TrkA and NRP1 were localized in mouse and human dorsal root ganglia (DRG) by immunofluorescence and RNAScope in situ hybridization. In mice, immunoreactive TrkA was detected in vesicles and immunoreactive NRP1 was localized at the plasma membrane of the same neurons (Figure 2A). *Ntrk1* (TrkA) and *Nrp1* (NRP1) mRNAs also colocalized in mouse neurons, identified by NeuN immunostaining (Figure 2B). *Nrp1* was detected in 42% of small-diameter nociceptors that expressed immunoreactive calcitonin gene-related peptide (CGRP) (Figure 2C). Immunoreactive NRP1 was also detected in satellite glial cells, identified by immunostaining for glutamine synthetase (GS) (Figure 2D). In humans, *NTRK1* and *NRP1* mRNAs colocalized in neurons (Figure 2E). *NRP1* was detected in 59% of human neurons expressing CGRP and 42% of neurons expressing P2X purinoceptor 3 (P2X3) (Figure 2F). In mice, *Nrp1* mRNA was expressed in 33% of *Ntrk1-*positive neurons (Figure 2G). In humans, *NRP1* was coexpressed in 93% of *NTRK1-*positive neurons (Figure 2H). Thus, TrkA and NRP1 are coexpressed in mouse and human DRG neurons. NRP1 is appropriately located to control NGF/TrkA signaling.

### NRP1 inhibitors suppress NGF-induced sensitization of TRPV1 in mouse DRG neurons.

**Figure 3:**
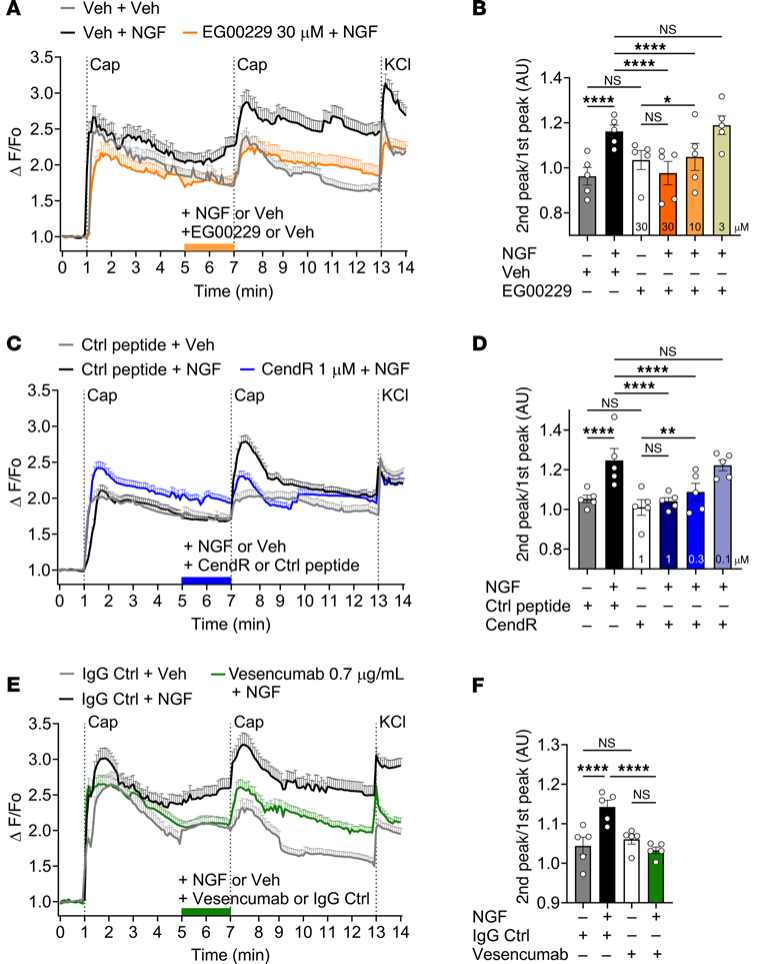
NRP1 inhibition prevents NGF-induced sensitization of TRPV1. (**A**, **C**, and **E**) Time course of responses of mouse DRG neurons to repeated challenge with capsaicin (Cap, 100 nM) expressed as ΔF/Fo ratio. (**B**, **D**, and **F**) Summary of responses to capsaicin expressed as the ΔF/Fo of the second capsaicin Ca^2+^ response over the ΔF/Fo of the first capsaicin Ca^2+^ response (2nd peak/1st peak). (**A** and **B**) Effect of NRP1 inhibitor EG00229 (3, 10, 30 μM, 30 minutes preincubation) or vehicle (Veh). (**A**) *n* = 64–119 cells per trace. (**B**) Summary from *n* = 5 independent experiments. (**C** and **D**) Effect of NRP1 inhibitor CendR or control (Ctrl) (0.1, 0.3, 1 μM). (**C**) *n* = 142–535 cells per trace. (**D**) Summary from *n* = 5 independent experiments. (**E** and **F**) Effect of a human mAb against the b1b2 domain of NRP1 (vesencumab) or control (Ctrl) IgG (0.7 μg/ml). (**E**) *n* = 97–199 cells per trace. (**F**) Summary from *n* = 5 independent experiments. A.U., arbitrary units. Data are represented as mean ± SEM. *P < 0.05; **P < 0.01; ****P < 0.0001. Two-way ANOVA with Tukey’s multiple-comparisons test.

Many algesic receptors, including TrkA, sensitize transient receptor potential vanilloid-1 (TRPV1) on nociceptors (8, 35). The contribution of NRP1 to NGF-induced sensitization of TRPV1 was determined by calcium imaging of mouse DRG neurons. Exposure of neurons to the TRPV1 agonist capsaicin (100 nM) increased [Ca^2+^]i by 239.3% ± 137.3% (mean ± SD, *n* = 1696 cells) of basal, consistent with TRPV1 activation (Figure 3A). The response to a second capsaicin challenge 6 minutes later was reduced to 96.13% ± 23.60% (*n* = 1696 cells) of the first response, indicating TRPV1 desensitization (*P* < 0.001, paired *t* test). When neurons were incubated with mouse NGF (100 nM) for 2 minutes before the second challenge, the response to the second capsaicin challenge was amplified to 119.3% ± 45.26% (*n* = 1621 cells) of the first response, denoting TRPV1 sensitization (*P* < 0.001, paired *t* test).

The contribution of NRP1 to NGF-induced sensitization of TRPV1 was examined by incubating DRG neurons with NRP1 antagonists or control reagents before the second capsaicin challenge. EG00229 is a small molecule inhibitor developed to inhibit binding of VEGF-A to the b1 domain of NRP1 (25). EG00229 (3, 10, 30 μM) caused a concentration-dependent inhibition of NGF-induced sensitization of TRPV1 compared with vehicle control (0.1% DMSO) (Figure 3, A and B). EG00229 30 μM prevented NGF-induced sensitization of TRPV1, 10 μM EG00229 partially inhibited TRPV1 sensitization, and 3 μM EG00229 was ineffective. To competitively inhibit binding of NGF to NRP1, a peptide fragment of NGF was synthesized that includes the 2 conserved CendR R/KXXR/K motifs within the C-terminus of NGF (underlined, QAAWRFIRIDTACVCVLSRKAVRRA, corresponds to 96–120 of mature NGF), which were predicted by molecular modeling to interact with the b1 domain of NRP1. A peptide fragment of NGF that was not predicted to interact with NRP1 (ARVAGQTRNITVDPRLFKKRRLRSP, corresponds to 61–85 of immature NGF) was used as a control (Ctrl peptide). The CendR peptide (0.1, 0.3, 1 μM) caused a concentration-dependent inhibition of NGF-induced sensitization of TRPV1 compared with a Ctrl peptide. CendR at 0.3 or 1 μM prevented TRPV1 sensitization whereas 0.1 μM CendR was ineffective (Figure 3, C and D). Vesencumab, a human mAb against the b1b2 domain of NRP1 (16) (0.7 μg/ml), prevented NGF-induced sensitization of TRPV1 compared with control IgG (Figure 3, E and F). None of the inhibitors affected the response to capsaicin in neurons that were not treated with NGF. The finding that 3 mechanistically distinct NRP1 inhibitors reproducibly blocked NGF-induced sensitization of TRPV1 suggests that NRP1 controls the pronociceptive actions of NGF and TrkA, potentially by enhancing the signaling competency of the NGF-TrkA complex that sensitizes TRPV1.

### NRP1 inhibitors suppress NGF-induced ionic currents in mouse and human DRG neurons.

**Figure 4:**
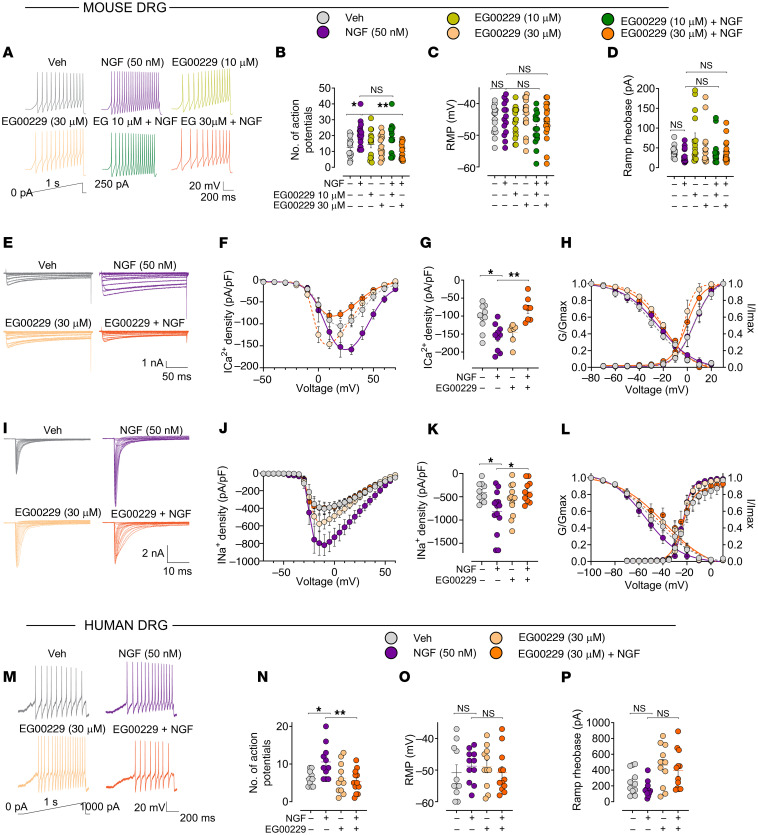
NRP1 inhibition prevents NGF-induced increases in neuronal firing, excitability, and ion channel currents. Effect of NRP1 inhibitor EG00229 (10 or 30 μM, 30 minutes) on responses of dissociated mouse (**A**–**L**) and human (**M**–**P**) DRG neurons to NGF (50 nM, 30 minutes). (**A**, **B**, **M**, and **N**) Representative action potential firing evoked by a depolarizing ramp stimulus (**A** and **M**), with summary of the number of evoked action potentials (**B** and **N**). *n* = 14–17 cells (**B**–**D**), *n* = 11–12 cells (**N**–**P**). (**C**, **D**, **O**, and **P**) Resting membrane potential (RMP) (**C**, **O**) and ramp rheobase (**D** and **P**). (**E**–**H**) Representative family of Ca^2+^ current traces recorded from small diameter DRG neurons in response to depolarization steps from –70 to +70 mV from a holding potential of –90 mV (**E**), with double Boltzmann fits for current density-voltage curve (**F**), summary of peak calcium current densities (**G**), and Boltzmann fits for voltage dependence of activation and inactivation (**H**). *n* = 7–10 cells. (**I**–**L**) Representative family of Na^+^ current traces, where currents were evoked by 150 ms pulses between −70 and +60 mV (**I**), with double Boltzmann fits for current density-voltage curve (**J**), summary of peak sodium current densities (**K**), and Boltzmann fits for voltage-dependence of activation and inactivation (**L**). *n* = 10–14 cells. Data are represented as mean ± SEM. *P < 0.05; ** *P* < 0.01. (**B**, **C**, **D**, **G**, **K**, **N**, **O**, and **P**) Kruskal-Wallis, Dunn’s multiple comparisons.

NGF/TrkA signaling promotes pain by kinase-mediated phosphorylation and sensitization of ion channels, leading to enhanced sensitivity to mechanical, thermal,and chemical stimuli, and by activating voltage-gated ion channels in DRG neurons (8). Patch-clamp recordings under current-clamp mode were made to determine whether NRP1 is necessary for NGF-induced excitability and ion channel activity in dissociated DRG neurons. Exposure of mouse DRG neurons to mouse NGF (50 nM) stimulated action potential firing of nociceptors elicited by a depolarizing ramp pulse (Figure 4, A and B). Preincubation with the NRP1 inhibitor EG00229 at 30 μM, but not 10 μM, prevented NGF-induced hyperexcitability (Figure 4, A and B). NGF or EG00229 did not affect the resting membrane potential (Figure 4C) or the rheobase, the minimum current necessary to elicit an action potential (Figure 4D). EG00229 at 30 μM was used in subsequent experiments.

Since both Ca^2+^ and Na^+^ channels control nociceptor excitability, we tested to determine whether NGF increased the activity of voltage-gated ion channels by making patch-clamp recordings under the voltage-clamp mode in dissociated mouse DRG. NGF (50 nM) increased total Ca^2+^ currents (Figure 4E) and current density (Figure 4, F and G) by approximately 50% when compared with vehicle (DMSO). EG00229 prevented NGF-induced activation of Ca^2+^ currents, but had no effect on Ca^2+^ currents in unstimulated DRGs (Figure 4, F and G). Half-maximal activation and inactivation (*V_1/2_*) potential, as well as slope factor values (*k*) for activation and inactivation were similar between the conditions tested, except for an approximately 8 mV hyperpolarizing shift in Ca^2+^ channel activation induced by EG00229 when compared with DMSO- and NGF-treated DRGs (Figure 4H and Supplemental Table 2). Similarly, EG00229 normalized NGF-induced increases in Na^+^ currents (Figure 4I). NGF caused a 2-fold increase in Na^+^ current density (Figure 4J) and peak current density (Figure 4K) when compared with vehicle-treated (DMSO-treated) DRG neurons. EG00229 abolished these effects of NGF but had no effects on Na^+^ currents in unstimulated DRGs (Figure 4, J and K). There were no detectable changes in the voltage dependence of activation and inactivation between the conditions tested, except for an approximately 10 mV depolarizing shift in the *V_1/2_* of inactivation of EG00229 and NGF-treated cells compared with control (Figure 4L). These data implicate voltage-gated Ca^2+^ and Na^+^ channels as downstream effectors of NRP1-mediated NGF signaling.

To assess human translation, the contribution of NRP1 to NGF-stimulated activation of human DRG neurons was studied. Human NGF (50 nM) increased the number of action potentials elicited by a depolarizing ramp pulse (Figure 4, M and N). Preincubation with the NRP1 inhibitor EG00229 (30 μM) (25) prevented NGF-induced hyperexcitability (Figure 4, M and N). NGF or EG00229 did not affect the resting membrane potential (Figure 4O) or the rheobase (Figure 4P). Thus, NRP1 facilitates NGF-induced sensitization of nociceptors in rodents to humans.

### NRP1 inhibitors suppress NGF-induced nociception in mice.

**Figure 5:**
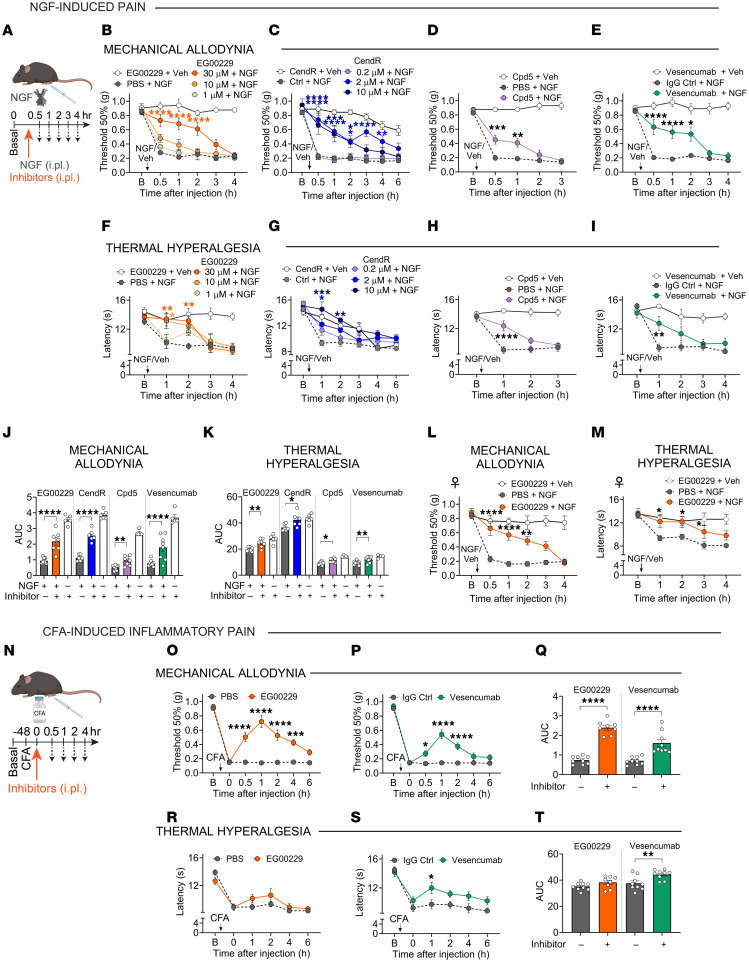
NRP1 inhibition abrogates NGF- and CFA-induced pain in mice. (**A**–**M**) NGF-induced pain in male (**B**–**K**) and female (**L** and **M**) mice. Effects of NRP1 inhibitors EG00229 (**B** and **F**; 1, 10, 30 μM/10 μl i.pl.), CendR (**C** and **G**; 0.2, 2 and 10 μM/10 μl i.pl.), compound 5 (**D** and **H**; Cpd5; 30 μM/10 μl i.pl.) or an antibody against the b1 domain of NRP1, vesencumab (**E** and **I**; 7 μg/10 μl i.pl.) in male mice. After baseline (B) measurements, inhibitors were coinjected with mouse NGF (50 ng/10 μl, i.pl.). Mechanical allodynia (**B**–**E**) and thermal hyperalgesia (**F**–**I**) were measured. *n* = 5–8 male mice per group. (**J** and **K**) AUC of EG00229 (30 μM/10 μl), CendR (2 μM/10 μl), compound 5 (30 μM/10 μl), and vesencumab (7 μg/10 μl) time courses. (**L** and **M**) Effects of NRP1 inhibitor EG00229 (30 μM/10 μl i.pl.) on NGF-induced nociception in female mice. Mechanical allodynia (**L**) and thermal hyperalgesia (**M**) were measured. *n* = 5–8 female mice per group. (**N**–**T**) CFA-induced inflammatory pain. Effects of EG00229 (**O** and **R**, 30 μM/10 μl) or vesencumab (**P** and **S**, 7 μg/10 μl). Inhibitors were injected (i.pl.) 48 hours after CFA (i.pl.). (**Q** and **T**) AUC of time courses. Mechanical allodynia (**O**–**Q**) and thermal hyperalgesia (**R**–**T**) were measured. *n* = 8–9 mice per group. Data are represented as mean ± SEM. *P < 0.05; **P < 0.01; ***P < 0.001; ****P < 0.0001 vs. PBS, control (Ctrl) peptide, or IgG Ctrl. (**B**–**I**, **L**, **M**, **O**, **P**, **R**, and **S**) Two-way ANOVA, Sídák’s multiple comparisons. (**J**, **K**, **Q**, and **T**) One-way ANOVA, Dunnett multiple comparisons.

NGF injection induces pain in humans and rodents (8). To test whether NRP1 is necessary for NGF-induced nociception, mouse NGF (50 ng, 10 μl) and NRP1 inhibitors or control reagents were administered to the hind paws of male mice by intraplantar (i.pl.) coinjection (Figure 5A). Withdrawal responses of injected (ipsilateral) hind paws to stimulation with von Frey filaments (VFF) and radiant heat were measured to evaluate mechanical allodynia and thermal hyperalgesia, respectively. NGF decreased the withdrawal threshold to VFF stimulation and reduced the latency of withdrawal to thermal stimulation within 30 minutes for at least 4 hours, indicating mechanical allodynia and thermal hyperalgesia (Figure 5, B–I). EG00229 (1, 10, or 30 μM/10 μl) (25) dose dependently inhibited NGF-induced mechanical allodynia and thermal hyperalgesia compared with vehicle control (PBS) (Figure 5, B and F, and Supplemental Figure 2, A and B). EG00229 at 30 μM strongly inhibited mechanical allodynia and thermal hyperalgesia for 2 hours, whereas 10 μM EG00229 inhibited only thermal hyperalgesia at 1 hour, and 1 μM EG00229 had no effect. CendR (0.2, 2, 10 μM/10 μl) also dose dependently inhibited NGF-induced mechanical allodynia and thermal hyperalgesia compared with control (Ctrl) peptide (Figure 5, C and G, and Supplemental Figure 2, C and D). CendR at 10 μM strongly inhibited mechanical allodynia and thermal hyperalgesia for 2 hours and 2 μM CendR inhibited mechanical allodynia for 4 hours and thermal hyperalgesia for 1 hour, whereas 0.2 μM CendR had no effect. Compound 5 (Cpd-5; 30 μM/10 μl), which like EG00229 blocks VEGF-A interaction with NRP1 (36), inhibited NGF-induced mechanical allodynia and thermal hyperalgesia for 1 hour compared with vehicle control (Figure 5, D and H). Vesencumab (7 μg/10 μl) (16) inhibited NGF-induced mechanical allodynia for 2 hours and thermal hyperalgesia for 1 hour compared with IgG control (Figure 5, E and I). Measurement of the integrated withdrawal responses (AUC of time courses) confirmed the inhibitory actions of these 4 mechanistically distinct NRP1 inhibitors on NGF-induced nociception (Figure 5, J and K). None of the inhibitors affected the withdrawal responses of the ipsilateral hind paws to mechanical or thermal stimuli in mice that did not receive NGF (Figure 5, B–I). NGF injection into the hind paws of female mice caused a comparable degree of mechanical allodynia and thermal hyperalgesia as in male mice, and EG00229 similarly inhibited NGF-induced nociception in female and male mice (Figure 5, L and M).

NGF mediates inflammatory pain (8), providing an opportunity to determine whether NRP1 is necessary for the pronociceptive actions of endogenous NGF. Complete Freund’s adjuvant (CFA) (1 mg/ml, 10 μl, i.pl.) was injected into the hind paws of male mice. After 48 hours, EG00229 (30 μM/10 μl), vehicle (PBS, 10 μl, i.pl.), vesencumab mAb (7 μg/10 μl), or IgG Ctrl (7 μg/10 μl) was injected into the inflamed hind paw (Figure 5N). CFA induced sustained mechanical allodynia and thermal hyperalgesia (Figure 5, N–T). EG00229 reversed mechanical allodynia for 4 hours (Figure 5O) but did not affect thermal hyperalgesia (Figure 5R). Vesencumab reversed mechanical allodynia for 2 hours (Figure 5P) and thermal hyperalgesia for 1 hour (Figure 5S). Measurement of the integrated withdrawal responses confirmed these inhibitory effects (Figure 5, Q and T).

The finding that 4 NRP1 inhibitors suppress NGF-evoked nociception supports the hypothesis that NRP1 enhances the signaling competency of the NGF-TrkA complex that induces pain.

### NRP1 controls NGF and TrkA kinase signaling.

**Figure 6:**
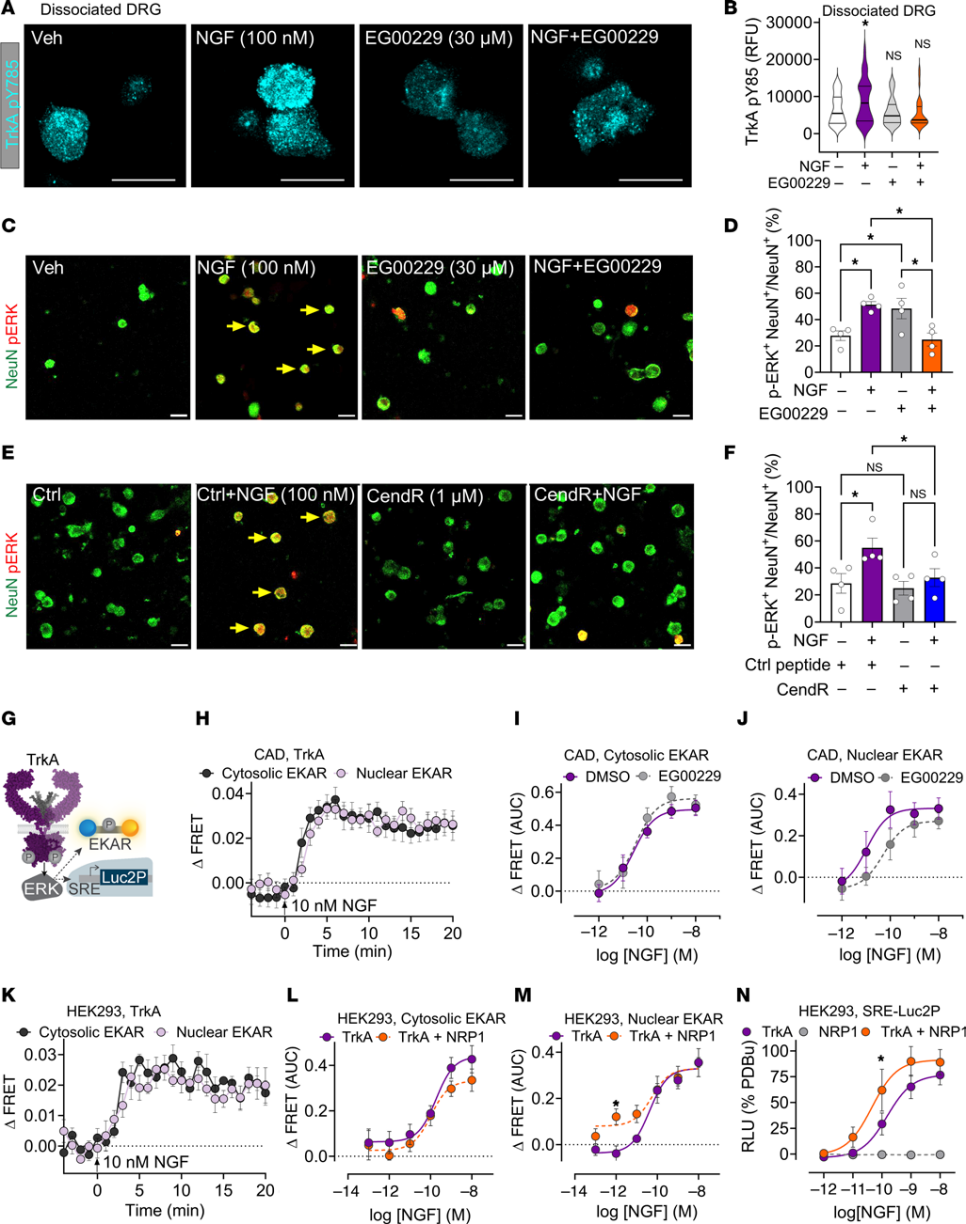
NRP1 modulates TrkA-mediated kinase signaling. (**A** and **B**) Effect of mouse NGF (100 nM, 15 minutes) and NRP1 inhibitor EG00229 (30 μM, 30 minutes preincubation) on phosphorylated TrkA Y785 staining in mouse DRG neurons. *n* = 34–44 neurons from 3 independent experiments. Scale bars: 20 μm. (**C**–**F**) Effect of mouse NGF (100 nM) and NRP1 inhibitors EG00229 (30 μM, 30 minutes preincubation) (**C** and **D**) and CendR (1 μM, 30 minutes preincubation) (**E** and **F**) on phosphorylated ERK Thr202/Tyr204 staining in mouse DRG neurons. *n* = 15–432 neurons from 4 independent experiments. Scale bars: 20 μm. (**G**–**N**) NGF-induced ERK signaling measured using FRET-based EKAR biosensors (**H**–**M**) or a downstream luciferase reporter (**N**). (**H**–**M**) NGF-induced modulation of ERK activity using biosensors localized to the cytosol (**H** and **I**) or nucleus (**H** and **J**) in neuron-like CAD cells expressing human TrkA. Kinetics of NGF-induced ERK monitored in CAD cells (**H**), comparing increasing NGF concentrations after preincubation with EG00229 (30 μM, 30 minutes) (**I**–**J**). (**K**–**M**) ERK signaling in HEK293T cells expressing TrkA alone or expressing both TrkA and NRP1 (**L** and **M**). (**N**) Effect of increasing NGF concentrations on ERK transcription in cells expressing TrkA, NRP1, or both (% positive control, 10 μM PDBu). RFU, relative fluorescence units. Data from 4–8 independent experiments with triplicate wells. Data are represented as mean ± SEM. *P < 0.05. (**B**, **M**, and **N**) One-way ANOVA, Sídák’s multiple comparisons. (**D** and **F**) Two-way ANOVA, Tukey’s multiple comparison.

NGF binding to extracellular immunoglobulin-like domains of TrkA induces conformational changes throughout the receptor dimer that initiate auto- and transphosphorylation of intracellular tyrosine residues (e.g., Y490, Y785) (29). To determine whether NRP1 controls NGF-stimulated phosphorylation of TrkA, a proximal and necessary component of TrkA signaling, dissociated mouse DRG neurons were stained with a phospho-specific antibody to TrkA Y785 (equivalent to Y791 in human TrkA). NGF (100 nM, 15 minutes) stimulated an approximately 1.4-fold increase in the intensity of TrkA phospho-Y785 immunofluorescence compared with vehicle (Figure 6, A and B). Preincubation with EG00229 (30 μM, 30 minutes) prevented NGF-stimulated phosphorylation of TrkA but had no effect on the basal unstimulated phosphorylation. These results support the hypothesis that NRP1 is necessary for NGF-induced activation of TrkA.

Activated TrkA stimulates extracellular signal-regulated kinase (ERK) phosphorylation, as well as AKT and phospholipase Cγ. In addition to triggering neuronal development, TrkA-induced ERK signaling contributes to NGF-induced sensitization of nociceptors and pain (8). To determine whether NRP1 is necessary for NGF-induced activation of ERK, dissociated mouse DRG neurons were coincubated with NGF (100 nM, 30 minutes) or vehicle together with inhibitors of NRP1 or control reagents. Cultures were stained with antibodies to phosphorylated T202/Y204 ERK1/2 and to the neuronal marker NeuN. NGF stimulated an approximately 1.8-fold increase in the number of neurons expressing phosphorylated ERK1/2 (Figure 6, C–F, yellow arrows). EG00229 (30 μM, 30 minutes) or CendR (1 μM, 30 minutes) prevented NGF-stimulated phosphorylation of ERK1/2 in neurons (Figure 6, D and F). Thus, NRP1 is necessary for NGF stimulation of ERK1/2 activity in DRG neurons.

The contribution of NRP1 to ERK activation was monitored in CAD cells, a neuron-like cell line modified from the Cath.a catecholaminergic cell line obtained from a mouse tumor (37). While CAD cells lack TrkA expression, they express p75^NTR^ and NRP1 (Supplemental Figure 3). To probe NGF/TrkA ERK signaling with high spatial and temporal resolution, Förster resonance energy transfer (FRET) extracellular signal-regulated kinase activity reporter (EKAR) biosensors targeted to the cytosol or nucleus were coexpressed with human TrkA in CAD cells. EKAR biosensors contain a reversible substrate sequence separated by 2 fluorophores (Figure 6G). NGF activated ERK in the cytosol and nucleus within 5 minutes for at least 20 minutes (Figure 6H). NGF-induced ERK activation was concentration dependent, with a higher potency for nuclear than cytosolic ERK (Figure 6, I and J, and Supplemental Table 3). Preincubation with EG00229 (30 μM) did not affect NGF activation of cytosolic ERK (Figure 6I), but significantly reduced the potency for NGF activation of nuclear ERK (Figure 6J and Supplemental Table 3; NGF EC_50_: DMSO control, ~8 pM; EG00229 ~98 pM; paired *t* test, *P* = 0.023).

The contribution of NRP1 to NGF-induced ERK signaling was further investigated by NRP1 overexpression with TrkA in HEK293T cells, which express low endogenous levels of these proteins. NGF activated ERK in the cytosol and nucleus of HEK293T cells (Figure 6K). While NRP1 coexpression did not influence cytosolic ERK signaling (Figure 6L), NRP1 expression significantly enhanced activation of nuclear ERK in response to low (1 pM) NGF concentrations (Figure 6M). The outcomes of nuclear ERK signaling were further studied by expression of a transcriptional luciferase reporter, where a luminescent protein is produced downstream of an ERK promoter (Figure 6G). NGF stimulated concentration-dependent ERK transcriptional activity with significantly higher potency in cells overexpressing TrkA and NRP1 compared with cells expressing TrkA alone (Figure 6N and Supplemental Table 3; NGF EC_50_: TrkA alone, 214 pM; TrkA + NRP1, 71 pM; paired *t* test, *P* = 0.0004). NGF did not stimulate ERK transcriptional activity in cells expressing NRP1 alone, confirming lack of inherent signaling capability of NRP1 (Figure 6N). These results support the notion that NRP1 enhances NGF-induced TrkA activation and signaling by pathways that underpin nociception. We next sought to determine the mechanism of NRP1-mediated potentiation of NGF/TrkA signaling.

### NRP1 is a chaperone that forms a complex with TrkA to control trafficking from the biosynthetic pathway to the plasma membrane.

**Figure 7:**
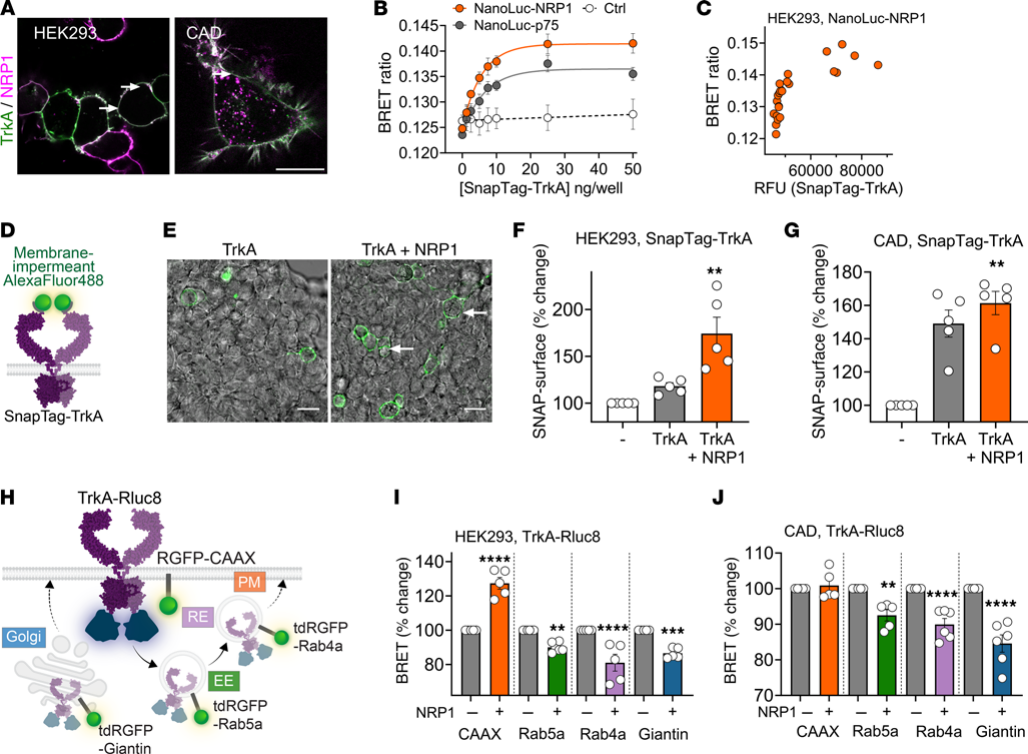
TrkA and NRP1 form a heteromeric complex. (**A**) HEK293T or CAD cells expressing SnapTag-TrkA and HaloTag-NRP1 simultaneously labeled with membrane-impermeant substrate (SNAPTag-Alexa Fluor 488, HaloTag-Alexa Fluor 660). Representative images from *n* = 5 independent experiments. (**B** and **C**) BRET assays to monitor proximity between NanoLuc-NRP1 or NanoLuc-p75^NTR^ and increasing SnapTag-TrkA DNA. Negative control, NanoLuc-TrkA, and SnapTag-CALCRL. Representative replicate (**C**) plotting BRET against RFUs. (**D**–**G**) Cell-surface TrkA in HEK293T imaged in the absence or presence of NRP1 (**E**). (**F** and **G**) Quantified fluorescence without receptor (–), SnapTag-TrkA alone, or SnapTag-TrkA cotransfected with NRP1 in HEK293T (**F**) or CAD (**G**) cells. (**H**–**J**) BRET between TrkA tagged with *Renilla* luciferase (Rluc8) and RGFP tagged markers of the plasma membrane (PM, RGFP-CAAX), early endosome (EE, tdRGFP-Rab5a), recycling endosomes (RE, tdRGFP-Rab4a), or the cis-Golgi apparatus (tdRGFP-Giantin). HEK293T cells (**I**) or CAD cells (**J**) were transfected with TrkA-Rluc8 in the absence (–) or presence (+) of NRP1. BRET was normalized relative to TrkA-Rluc8 alone (100%). Scale bars: 20 μm. Data from 5–6 independent experiments with triplicate wells. Data are represented as mean ± SEM. **P < 0.01; ***P < 0.001; ****P < 0.0001. (**F** and **G**) Paired *t* test. (**I** and **J**) One-way ANOVA with Šídák’s multiple comparisons.

In addition to directly binding NGF, NRP1 could amplify NGF/TrkA signaling by receptor/coreceptor interactions with TrkA, akin to its coreceptor function with VEGFR2 (38). Cell-surface expression of human TrkA and NRP1 was measured by specific substrate-based labeling of enzymatic tags genetically fused to the extracellular N-terminus of either receptor. SnapTag-TrkA and HaloTag-NRP1 were expressed in HEK293T and CAD cells and covalently labeled with membrane-impermeant Alexa Fluor substrates, thereby selectively labeling receptors transported to the cell surface. SnapTag-TrkA and HaloTag-NRP1 were highly colocalized at the cell surface of HEK293T and CAD cells, with the latter also showing a high level of colocalization in subcellular compartments (Figure 7A). Labeling specificity was confirmed in cells expressing TrkA or NRP1 alone (Supplemental Figure 4).

To investigate the formation of a heteromeric complex between TrkA and NRP1, BRET was measured in HEK293T cells expressing NanoLuc-NRP1 and SnapTag-TrkA. Extracellular N-terminal NanoLuc acts as an energy donor to excite a nearby SnapTag. Measurement of BRET between NanoLuc-NRP1 and increasing levels of SnapTag-TrkA revealed a hyperbolic relationship, indicative of assembly of a heteromeric complex between NanoLuc-NRP1 and SnapTag-TrkA (Figure 7, B and C). As a positive control, a hyperbolic BRET signal relationship was also detected between NanoLuc-p75^NTR^, which is known to interact with TrkA (8), and increasing levels of SnapTag-TrkA. In contrast, there was a linear BRET signal between NanoLuc-NRP1 and increasing levels of SnapTag-calcitonin-like receptor (CALCRL), an unrelated transmembrane receptor for CGRP. To verify whether this relationship was observed with protein expression, data from a representative experiment quantifying cell-surface SnapTag labeling also demonstrated a hyperbolic curve between TrkA and NRP1 (Figure 7C). These results suggest that NRP1 and TrkA colocalize at the plasma membrane as a heteromeric complex.

Recruiting TrkA to the plasma membrane would enable increased access to extracellular NGF. The effect of NRP1 on the cell-surface expression of TrkA was determined using the membrane-impermeant SnapTag fluorophore to selectively label cell surface TrkA (Figure 7D). NRP1 coexpression increased levels of TrkA at the surface in HEK293T cells by 150% ± 43% and of CAD cells by 109% ± 4% (Figure 7, E–G). Enhanced bystander BRET, which capitalizes on the endogenous affinity of pairs of *Renilla*-tagged proteins to boost sensitivity (39), was used to quantify the effects of NRP1 expression on the localization of TrkA in different subcellular compartments of living cells. TrkA tagged on the C-terminus with Renilla luciferase (Rluc8, BRET) was coexpressed in HEK293T or CAD cells with proteins resident of the plasma membrane (CAAX), early endosomes (Rab5a), recycling endosomes (Rab4a), and the cis-Golgi apparatus (Giantin) tagged with *Renilla* green fluorescent protein (RGFP) (Figure 7H). In HEK293T cells, NRP1 expression significantly increased BRET between TrkA-Rluc8 and RGFP-CAAX and significantly decreased BRET between TrkA-Rluc8 and tdRGFP-Rab5a, tdRGFP-Rab4a, and tdRGFP-Giantin (Figure 7I). In CAD cells, NRP1 expression did not affect BRET between TrkA-Rluc8 and RGFP-CAAX, but significantly decreased BRET between TrkA-Rluc8 and tdRGFP-Rab5a, tdRGFP-Rab4a, and tdRGFP-Giantin (Figure 7J). These results indicate that NRP1 expression causes a redistribution of TrkA from subcellular regions involved in receptor recycling or de novo export to the plasma membrane, consistent with a chaperone function. Control studies confirmed NRP1 was successfully expressed using a HaloTag label (Supplemental Figure 5, A and B). While there was a small reduction in TrkA-Rluc8 expression in CAD cells upon NRP1 coexpression, NRP1 coexpression had no significant effect on overall TrkA expression in HEK293T cells (Supplemental Figure 5, C and D). These results suggest that NRP1 acts as a chaperone for TrkA.

### NRP1 enhances NGF-induced TrkA trafficking and dimerization.

**Figure 8:**
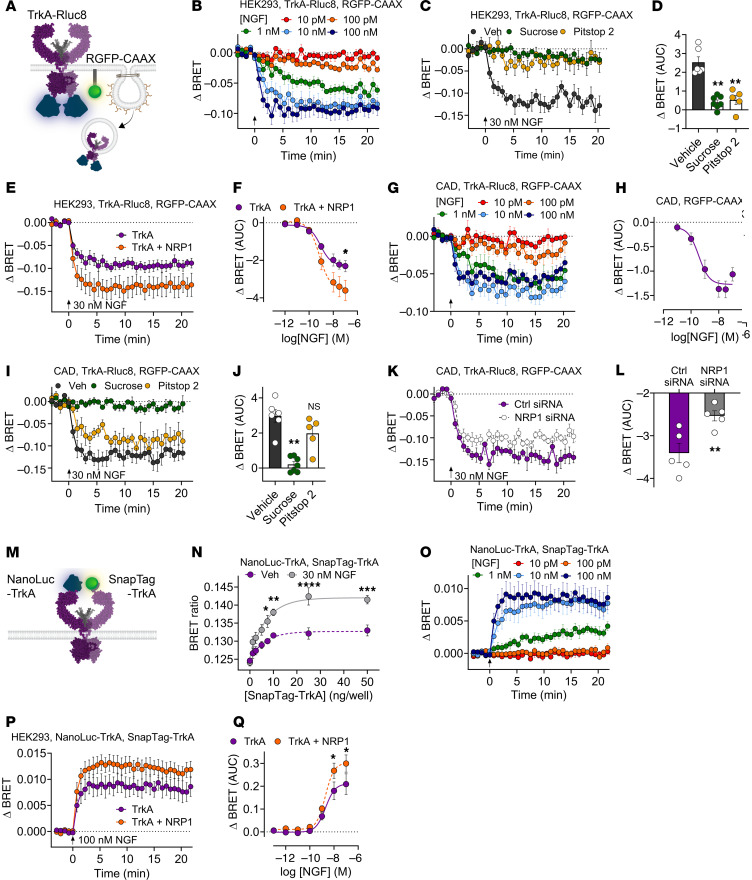
NRP1 modulates NGF-induced TrkA trafficking and RTK oligomerization. (**A**–**L**) BRET measurements between Rluc8-tagged TrkA and a fluorescent marker of the plasma membrane (RGFP-CAAX) at 37°C. Decreased BRET indicates TrkA-Rluc8 removal from the plasma membrane (**A**). (**B**–**F**) Trafficking in HEK293T cells incubated with graded concentrations of NGF (**B**) or preincubated for 30 minutes with hypertonic sucrose (0.45 M), clathrin inhibitor pitstop 2 (30 μM), or vehicle (Veh) (**C** and **D**) before NGF stimulation. Effect of NRP1 coexpression on NGF-stimulated endocytosis of TrkA (**E** and **F**). (**G**–**L**) Trafficking in CAD cells expressing human TrkA incubated with graded concentrations of NGF (**G** and **H**), or preincubated for 30 minutes with hypertonic sucrose (0.45 M), clathrin inhibitor pitstop 2 (30 μM), or vehicle (Veh) (**I** and **J**) before NGF stimulation, or transfected with control or mouse NRP1 siRNA (**K** and **L**). (**M**–**Q**) BRET measurements between NanoLuc-TrkA and SnapTag-TrkA to assess TrkA oligomerization in HEK293T cells. (**N**) BRET with increasing expression of SnapTag-TrkA and fixed NanoLuc-TrkA (10 ng), after 30 minutes incubation with vehicle or NGF. (**O**–**Q**) Using a fixed donor:acceptor ratio (1:2.5), oligomerization kinetics at 37ºC in response to increasing NGF concentrations (**O**) in the absence and presence of NRP1 coexpression (**P** and **Q**; showing the same 100 nM NGF kinetic data for TrkA in **O** and **P**). Data from 4–6 independent experiments with triplicate wells. Data are represented as mean ± SEM. *P < 0.05, **P < 0.01, ***P < 0.001, ****P < 0.0001. (**G**) Unpaired *t* test. (**D**, **I**, and **K**) One-way ANOVA with Sídák’s multiple comparisons.

Upon NGF stimulation, TrkA traffics to endosomes by clathrin-dependent and -independent mechanisms. NGF/TrkA signalosomes are retrogradely transported in endosomes or multivesicular bodies to the soma of sympathetic neurons, where they mediate the neurotrophic actions of NGF (3, 4). To determine the effects of NRP1 on NGF-evoked endocytosis of TrkA, BRET was measured between TrkA-Rluc8 and plasma membrane marker RGFP-CAAX (Figure 8A). NGF caused a concentration-dependent decrease in TrkA-Rluc8 and RGFP-CAAX BRET (EC_50_ ~870 pM), with 10–100 nM NGF inducing a maximal decrease within 5 minutes that was sustained for 20 minutes, consistent with TrkA endocytosis (Figure 8B and Supplemental Table 3). Hypertonic (0.45 M) sucrose or the clathrin inhibitor pitstop 2 (30 μM) prevented NGF-induced endocytosis of TrkA (Figure 8, C and D). Coexpression of NRP1 with TrkA enhanced NGF-stimulated endocytosis of TrkA (Figure 8, E and F). This effect of NRP1 was observed at higher NGF concentrations (>10 nM), with minimal effect on the potency of NGF-induced endocytosis of TrkA (Supplemental Table 3). NGF also stimulated concentration-dependent removal of TrkA from the plasma membrane of CAD cells (EC_50_ ~490 nM; pEC_50_ = 9.31 ± 0.18, *n* = 4) that was inhibited by hypertonic sucrose and pitstop 2 (Figure 8, G–J). Knockdown of endogenous NRP1 with siRNA significantly inhibited NGF-induced endocytosis of TrkA in CAD cells (Figure 8, K and L). NRP1 knockdown was confirmed at a protein level using immunofluorescence in CAD cells, while there was no effect on TrkA expression (Supplemental Figure 5, E and F). Thus, whereas NRP1 overexpression enhances agonist-stimulated endocytosis of TrkA in HEK293T cells, NRP1 knockdown has the opposite effect in CAD cells. The results are consistent with a chaperone role for NRP1 in NGF-stimulated endocytosis of TrkA.

Dimerization of TrkA is pivotal for NGF/TrkA signaling (8). The contribution of NRP1 to TrkA oligomerization was evaluated by measuring BRET between NanoLuc-TrkA and SnapTag-TrkA in HEK293T cells (Figure 8M). Expression of increasing amounts of SnapTag-TrkA in HEK293T cells expressing a fixed amount of NanoLuc-TrkA produced a hyperbolic BRET signal, indicating oligomerization (Figure 8N). NGF (30 nM) significantly enhanced this signal, consistent with agonist-evoked TrkA oligomerization. In cells expressing a fixed ratio of SnapTag-TrkA and NanoLuc-TrkA, NGF stimulated a concentration-dependent increase in BRET that was maximal after 5 minutes and sustained for at least 20 minutes (EC_50_ 2.3 nM; Figure 8O and Supplemental Table 3). While NGF had a slightly lower potency with respect to dimerization than endocytosis, the kinetics of TrkA dimerization and endocytosis were similar. NRP1 overexpression enhanced TrkA oligomerization in response to higher NGF concentrations (>10 nM) (Figure 8, P and Q). As dimerization is the first step in RTK activation, with well-established evidence for TrkA signaling from endosomes, these results provide a mechanistic insight into the role of NRP1 in controlling TrkA signaling and trafficking.

### GIPC1 mediates NRP1/TrkA interactions and NGF-induced pain signaling.

**Figure 9:**
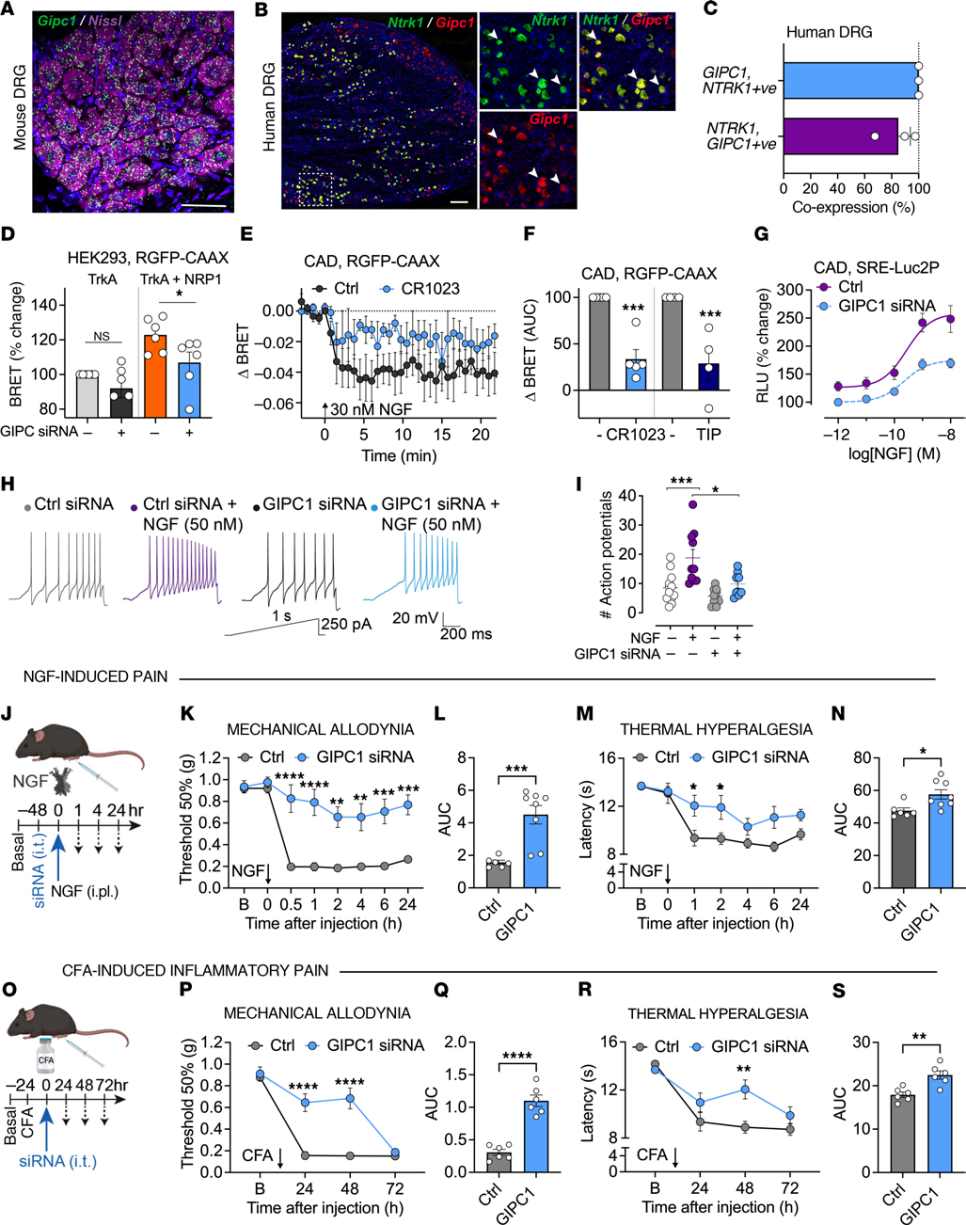
GIPC1 modulates TrkA trafficking, signaling, and NGF-induced nociception. (**A**–**C**) RNAScope localization of *Gipc1* mRNA in mouse DRG (**A**) and of *NTRK1* and *GIPC1* mRNA in human DRG (**B**). Arrows indicate mRNA expression within the same cell. Representative images, *n* = 5 mice and *n* = 3 humans. Scale bars: 500 μm. (**C**) Percentage of human DRG neurons expressing *NTRK1* or *GIPC1* that coexpress *GIPC1* or *NRP1*. Hybridized positive neurons (%) from *n* = 3 humans. (**D**) Effect of GIPC1 siRNA on BRET measurements of TrkA levels at the plasma membrane of HEK293T cells under basal conditions and after coexpression with NRP1. (**E** and **F**) Effect of 30 minutes preincubation of GIPC1 antagonist (300 μM CR1023 or inactive control, Ctrl) or myosin VI inhibitor (50 μM 2,4,6-triiodophenol, TIP) on NGF-induced TrkA-Rluc8 trafficking from a marker of the plasma membrane (RGFP-CAAX) in CAD cells. (**G**) Effect of GIPC1 siRNA on NGF-induced downstream ERK transcription in CAD cells. Data from 5–6 independent experiments with triplicate wells. (**H** and **I**) Sample traces of action potential firing in mouse DRG neurons evoked by injecting a 1-second ramp pulse from 0 to 250 pA (**G**), with the number of evoked action potentials (**H**). *n* = 7–10 cells. (**J**–**N**) NGF-induced pain. Effects of GIPC1 or Ctrl siRNA (i.t.) on NGF-induced (50 ng/10 μl, i.pl.) mechanical allodynia (**K** and **L**) and thermal hyperalgesia (**M** and **N**) in the ipsilateral paw. (**L** and **N**) AUC of time courses. (**O**–**S**) CFA-induced pain. Effects of GIPC1 or Ctrl siRNA (i.t.) on CFA-induced (i.pl.) mechanical allodynia (**P** and **Q**) and thermal hyperalgesia (**R** and **S**). (**Q** and **S**) AUC of time courses. *n* = 6–8 mice per group. B, basal. Data are represented as mean ± SEM. *P < 0.05; **P < 0.01; ***P < 0.001; ****P < 0.0001. (**D** and **F**) One-way ANOVA, Sídák’s multiple comparisons. (**I**) Tukey’s multiple comparison. (**K**, **M**, **P**, and **R**) Two-way ANOVA, Sídák’s multiple comparisons. (**L**, **N**, **Q**, and **S**) Unpaired *t* test.

While NRP1 lacks intrinsic catalytic activity, the short cytoplasmic C-terminus interacts with G α interacting protein C-terminus 1 (GIPC1), or synectin, through a PDZ domain (40). GIPC1 also interacts with the membrane-proximal regions of TrkA (41). Thus, interaction with GIPC1 could underpin the coreceptor function of NRP1 in NGF/TrkA-evoked nociception. Analysis by RNAScope in situ hybridization revealed that *Gipc1* mRNA was expressed by all mouse DRG neurons (Figure 9A). In humans, *GIPC1* was expressed by 100% of *NTRK1*-positive neurons and *NTRK1* was expressed by 85% of *GIPC1*-positive neurons (Figure 9, B and C).

GIPC1 is an intracellular adaptor protein that associates with receptors and channels to regulate their trafficking by interacting with the inwardly directed myosin VI motor (42). To determine whether GIPC1 is necessary for the TrkA chaperone function of NRP1, BRET between TrkA-Rluc8 and RGFP-CAAX was measured in HEK293T cells after GIPC1 knockdown. GIPC1 siRNA inhibited NRP1-induced plasma membrane expression of TrkA (Figure 9D). In CAD cells, preincubation with a GIPC1 inhibitor (300 μM CR1023; ref. 43) or a myosin VI inhibitor (50 μM TIP; ref. 44) reduced NGF-induced TrkA trafficking to the plasma membrane (Figure 9, E and F). Similarly, GIPC1 siRNA inhibited the maximal response of NGF-induced ERK signaling quantified using the downstream transcriptional reporter in CAD cells, with no effect on potency (Figure 9G) (Ctrl siRNA pEC_50_ = 9.58 ± 0.03, GIPC1 siRNA pEC_50_ = 9.63 ± 0.09, *n* = 5). GIPC1 siRNA also prevented NGF-induced action potential firing in mouse DRG nociceptors, determined by patch-clamp recordings (Figure 9, H and I). GIPC1 siRNA knockdown was confirmed at an mRNA level in HEK293T cells, CAD cells, and DRG (Supplemental Figure 6, A–C).

To evaluate the role of GIPC1 in NGF-induced nociceptive behavior, GIPC1 or control siRNA was administered to male mice by intrathecal (i.t.) injection 48 hours before NGF (50 ng/10 μl, i.pl.) (Figure 9J). In mice treated with control siRNA, NGF caused mechanical allodynia and thermal hyperalgesia in the ipsilateral paw within 30 minutes for at least 24 hours (Figure 9, K–N). GIPC1 siRNA prevented NGF-evoked mechanical allodynia for at least 24 hours and inhibited thermal hyperalgesia for 2 hours. NGF or siRNA administration did not affect withdrawal responses of the contralateral paw to mechanical stimuli (Supplemental Figure 7A). The nociceptive role of GIPC1 was also investigated in a preclinical model of inflammatory pain in male mice. Control or GIPC1 siRNA was administered (i.t.) 24 hours after CFA (1 mg/ml, 10 μl, i.pl.) (Figure 9O). In mice treated with control siRNA, CFA caused mechanical allodynia and thermal hyperalgesia for at least 72 hours (Figure 9, P–S). GIPC1 siRNA inhibited CFA-induced mechanical allodynia and thermal hyperalgesia after 24 and 48 hours. CFA or siRNA administration did not affect withdrawal responses of the contralateral paw to mechanical stimuli (Supplemental Figure 7B). GIPC1 siRNA caused knockdown of *Gipc1* mRNA in DRG, determined by RNAScope in situ hybridization (Supplemental Figure 6D). These results suggest that GIPC1 controls NGF-evoked pain.

## Discussion

**Figure 10:**
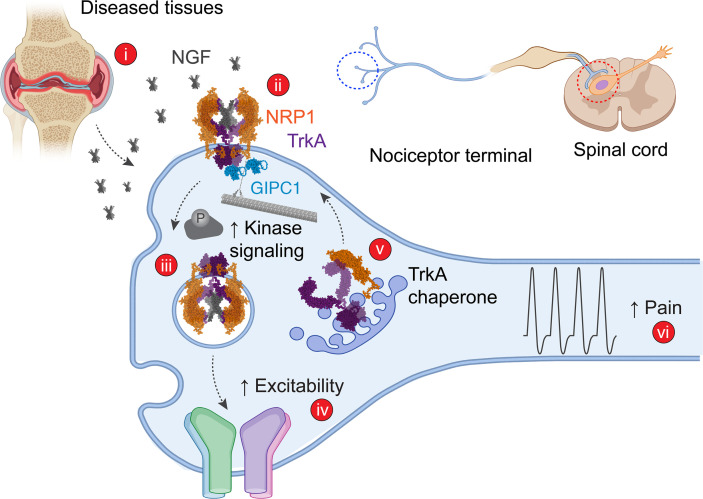
Hypothesized mechanism by which NRP1 mediates NGF/TrkA pain signaling. i. NGF is released from diseased tissues (e.g., sites of injury, inflammation, cancer) in close proximity to the peripheral endings of nociceptors. ii. At the surface of nociceptors, NGF binds to both NRP1 and TrkA, forming a ternary NGF/NRP1/TrkA complex with a 2:2:2 stoichiometry. iii. TrkA signals from the plasma membrane and endosomes to activate kinases and ion channels. iv. Activation and sensitization of TRPV1 and Na^+^ and Ca^2+^ channels lead to increased excitability of nociceptors. v. NRP1 chaperones TrkA from the biosynthetic pathway to the plasma membrane and to signaling endosomes, which further enhances excitability of nociceptors. vi. GIPC1 interacts with NRP1 and TrkA, linking the complex to the myosin VI molecular motor to amplify pain signaling. As such, by binding NGF and interacting with TrkA, NRP1 is a coreceptor that facilitates NGF/TrkA signaling of pain.

We report that NRP1 is a coreceptor for NGF and TrkA signaling of pain, demonstrated in rodent and human tissue. NRP1 inhibition attenuated NGF and TrkA signaling in cell lines and to block the pronociceptive actions of NGF in mice and isolated human and mouse nociceptors, whereas NRP1 overexpression amplified NGF and TrkA signaling and trafficking. In a similar manner, NRP1 is a coreceptor for VEGF-mediated angiogenesis, demonstrated using pharmacological (24) and genetic (45) interventions. Our results show that NRP1 promotes NGF/TrkA-mediated pain by at least 2 mechanisms: as a coreceptor that interacts with NGF and TrkA to form a ternary NGF/TrkA/NRP1 signaling complex and as a chaperone that enhances TrkA trafficking from the biosynthetic pathway to the plasma membrane and then signaling endosomes (Figure 10).

Several observations suggest that NRP1 is an NGF and TrkA coreceptor. *Nrp1*/NRP1 was coexpressed with *Ntrk1*/TrkA in nociceptors of mouse and human DRG. In support of these findings, a single-cell transcriptomics study reported coexpression of *Nrp1* and *Ntrk1* in C-fibers in mouse and human DRG, including 37% of CGRP-positive neurons (46). Thus, NRP1 is appropriately colocalized with TrkA in mouse and human nociceptors to control NGF and TrkA signaling of pain. In contrast to our findings, *Nrp1* and *Ntrk1* were not detected in satellite glial cells (46). This discrepancy, which may be attributable to different methodology, warrants further investigation. Although our results provide evidence for NRP1 and TrkA interactions within the same cell, the ECD of NRP1 could interact with the ECD of TrkA on adjacent cells. In a similar manner, NRP1 can interact with VEGFR2 both when they are coexpressed in the same cell, where the extracellular and intracellular domains associate, and in adjacent cells, where only the ECDs associate (47). This intercellular signaling may underlie functional interactions between nociceptors and adjacent cells (e.g., glial cells) that contribute to pain.

MST revealed that NGF and NRP1 interact with nanomolar affinity. BRET proximity assays provide evidence that NGF interacts with NRP1 at the cell surface. Future studies of the effects of NRP1 on NGF/TrkA binding kinetics are warranted because NRP1 potentiation of VEGFR2 signaling could be linked to growth factor binding kinetics (26). Measurement of BRET between NRP1 and increasing levels of TrkA revealed a hyperbolic relationship, indicative of assembly of a NRP1/TrkA complex. A computational docking protocol, which was based on the structure of the NGF/TrkA complex (29) and on structural features underlying interactions between NRP1 with other growth factors (20, 30–32), predicted the formation of a ternary NGF/TrkA/NRP1 complex at the cell surface with a 2:2:2 stoichiometry. This analysis predicts that R118 of the NGF CendR motif K_115_AVR_118_ binds to a C-terminal arginine–binding pocket of the NRP1 b1 domain through hydrogen bonding interactions between NGF R118 and NRP1 conserved residues Y297, D320, and Y353. The membrane proximal MAM domain of NRP1 is proposed to orient other extracellular NRP1 domains away from the plasma membrane, enabling 1 NRP1 molecule to interact with 1 TrkA molecule and the NGF dimer. Structural analysis of the putative NGF/TrkA/NRP1 complex will be required to confirm this prediction.

The proposed chaperone function of NRP1 is supported by BRET assays showing that NRP1 and TrkA form a heteromeric complex and that NRP1 expression reroutes TrkA from the biosynthetic pathway to the plasma membrane and subsequently to signaling endosomes. By chaperoning TrkA to the cell surface and then to signaling endosomes, NRP1 is expected to amplify the intracellular signaling of NGF/TrkA, which is known to regulate gene transcription (4, 48, 49). The observation that NRP1 expression potentiates NGF-stimulated TrkA dimerization, endocytosis, and kinase signaling is consistent with this role of NRP1. Further studies are required to experimentally confirm the potential NRP1/TrkA interaction sites predicted by molecular modeling to mediate the chaperone function of NRP1.

Although TrkA is the principal pronociceptive NGF receptor, p75^NTR^ also contributes to NGF-induced pain (50). Like NRP1, p75^NTR^ accelerates NGF binding and internalization when coexpressed with TrkA (51). Since the NGF/p75^NTR^-binding site (52) does not directly conflict with the proposed NGF/NRP1-binding site, a TrkA/p75^NTR^/NRP1 complex may also interact with NGF, although there could be steric clashes between NRP1 and p75^NTR^ for the same binding site on NGF. Further studies are required to determine whether NRP1 regulates NGF interaction with p75^NTR^. Despite the glycosylated nature of NRP1, indirect association of NGF with matrix components is unlikely to mediate NGF/NRP1 interactions, as NGF does not directly interact with the extracellular matrix (53).

Our results show that GIPC1 is a previously unrecognized mediator of pain. In the context of NGF/TrkA-evoked pain, GIPC1 interacts with TrkA (41) and NRP1 (40) and could thus scaffold TrkA/NRP1 interactions to facilitate NGF signaling. By coupling TrkA and NRP1 to the myosin VI molecular motor, GIPC1 may mediate trafficking of TrkA and NRP1 to the plasma membrane and signaling endosomes. In support of this possibility, GIPC1 disruption inhibited NRP1-stimulated translocation of TrkA to the plasma membrane and suppressed NGF-evoked endocytosis of TrkA and ERK signaling, in line with reports in other systems (54). GIPC1 prominently colocalized with NRP1 and thus TrkA in human and mouse nociceptors and is therefore appropriately located to control NGF-evoked pain. However, since GIPC1 regulates the trafficking of many receptors and ion channels, other mechanisms could also mediate the antinociceptive effects of GIPC1 knockdown in mice.

Electrophysiological and calcium imaging studies of human and mouse nociceptors and analysis of nociceptive behavior in mice support a role for NRP1 and GIPC1 in NGF-induced pain. NRP1 inhibitors (EG00229, CendR, compound 5, vesencumab) and GIPC1 siRNA suppressed NGF-evoked sensitization of mouse and human nociceptors and mechanical allodynia and thermal hyperalgesia in mice. EG00229 had similar effects on mouse and human nociceptors, which supports human translation. Although pharmacological inhibitors can lack selectivity and NRP1 deletion from nociceptors would unequivocally define its role in pain, the finding that mechanistically distinct inhibitors have similar effects provides confidence in specificity. EG00229 and compound 5 inhibit binding of VEGF-A to the b1 domain of NRP1 (25, 36). CendR corresponds to an NGF fragment that includes the 2 R/KXXR/K domains predicted to interact with the extracellular b domains of NRP1 and likely competes with NGF for NRP1 binding. Vesencumab is a human mAb against the b1b2 domain of NRP1 (16). These inhibitors all suppressed NGF-induced nociception.

NGF causes pain by temporally distinct mechanisms, including rapid sensitization of ion channels and sustained transcription of pronociceptive mediators (55). Our results suggest that NRP1 contributes to both the rapid (TrkA dimerization, phosphorylation, channel activation) and sustained (ERK activation, transcription) actions of NGF (Figure 10). The finding that vesencumab and CendR, which are unlikely to penetrate the plasma membrane, block NGF-evoked nociception, suggest that NRP1 binding to NGF and association with TrkA at the plasma membrane mediate the rapid pronociceptive actions of NGF. By chaperoning TrkA to the plasma membrane and enhancing trafficking of the NGF/TrkA complex to signaling endosomes, NRP1 and GIPC1 could contribute to sustained NGF-evoked nociception. NGF/TrkA signaling endosomes have been identified in sympathetic (49) and DRG (48) neurons, and retrograde NGF/TrkA signaling controls gene expression during the development of sympathetic neurons (49). Whether endosomal signaling of NGF/TrkA contributes to pain, as observed with G protein–coupled receptors (56), deserves further attention, including determination of the contribution of NRP1 to NGF-induced expression of pronociceptive transmitters and channels. NRP1 interacts with semaphorins (19) as a coreceptor for plexins, which regulate TrkA retrograde signaling (57). NRP1 localizes to axonal growth cones (58) and NGF-responsive DRG neurons during neurite sprouting (59). Thus, disruption of NRP1 could affect NGF-induced neuronal growth, with implications for the neuroplasticity associated with chronic pain.

Further studies are required to determine whether NRP1 inhibitors are a safe and effective treatment for pain. NGF is implicated in inflammatory, neuropathic, surgical, and cancer pain (8), highlighting the need to study contributions of NRP1 across pain pathologies. While NGF mAbs provided beneficial pain relief to patients with arthritis, rapidly progressing osteoarthritis in some patients precluded FDA approval (12). The mechanism responsible for osteoarthritis in patients treated with NGF mAbs is not understood, and whether NRP1 differentially regulates the pronociceptive and protective actions of NGF in joints is unknown. The finding that NRP1 is enriched with TrkA in nociceptors supports targeting NRP1 for the treatment of pain. Therapies targeting interactions among NGF, TrkA, and NRP1 in nociceptors, such as CendR peptides designed to block NGF and NRP1 association, may obviate the detrimental effects of global NGF sequestration with mAbs. TrkA and NRP1 are also expressed by chondrocytes, where NGF and TrkA have been implicated in articular cartridge homeostasis (60). Targeting Sema3A-NRP1 signaling has been proposed as a therapy for arthritis (61). The effects of NRP1 antagonism on joint health and disease warrants further study. Because NRP1 interacts with pronociceptive growth factors in addition to NGF, including VEGF-A (18), NRP1 inhibitors could suppress several forms of pain. In the context of cancer, NRP1 inhibitors would be expected to suppress NGF-evoked pain and VEGF-A–mediated angiogenesis in tumors, although impaired wound healing could be a liability. Although NRP1 mAbs were well tolerated in human subjects (62), analysis of side effects of NRP1 inhibition will be required to advance NRP1-directed therapies to the clinic. Identifying the antinociceptive efficacy of vesencumab highlights an opportunity to repurpose a biologic developed for cancer for the treatment of chronic pain, facilitating the development of a nonopioid therapeutic targeting NGF signaling through its coreceptor identified in this study.

## Methods

### See Supplemental Methods

#### Sex as a biological variable.

Nociception was studied in male and female mice.

#### MST.

Interaction between NGF and recombinant NRP1 was measured by MST.

#### HiBiT-BRET binding assays.

Interaction between NGF and NRP1 at the cell surface was measured using BRET. SnapTag-NRP1 or SnapTag-NRP1 Y297A was expressed in HEK293T cells and labeled with SNAP-Surface Alexa Fluor 488. IL-6-HiBiT-VEGF_165_a (positive control) or IL-6-NGF-HiBiT was expressed in other HEK293T cells. Secreted growth factor (cell supernatant) was incubated with recombinant HaloTag-LgBiT and furimazine. NRP1-expressing cells were preincubated with vehicle or unlabeled VEGF_165_a, followed by supernatant. Luminescence and fluorescence were recorded.

#### NGF/TrkA/NRP1 modeling.

Ternary complexes of human NGF/TrkA/NRP1 were generated through an information-driven computational docking protocol (27).

#### Immunostaining, RNAScope.

Human donor information has been provided (63). Human and mouse DRG (L4-L5) were fixed and sectioned. TrkA, NRP1, GS, CGRP, and P2X3 were detected by immunofluorescence. NRP1, TrkA, and GIPC1 were detected by RNAScope.

#### DRG culture.

Human DRG suspension cells were from AnaBios Corp. and were studied within 96 hours. Thoracic and lumbar mouse DRG were enzymatically dissociated and studied within 48 hours.

#### Nociceptor activation.

Action potentials were recorded from human and mouse DRG neurons in whole-cell patch-clamp configuration and current-clamp mode. Rheobase, Ca^2+^ currents, and Na^+^ currents were measured (17, 18). TRPV1 activity in mouse DRG neurons was determined by measuring capsaicin stimulation of [Ca^2+^]_i_ using Fluo-4AM. NGF-stimulated channel activity was determined in neurons preincubated with NRP1 inhibitors or vehicle, or GIPC1 or Ctrl siRNA.

#### Nociception.

NGF or CFA was injected (i.pl.) into the hind paws of mice. NRP1 inhibitors or vehicle was administered (i.pl.) concomitantly with NGF or 48 hours after CFA. Mechanical allodynia and thermal hyperalgesia were measured (63). Investigators were blinded to treatment. GIPC1 or Ctrl siRNA was injected (i.t., L4-L5) into mice 48 hours before NGF or 48 hours after CFA.

#### TrkA, ERK phosphorylation.

Activated TrkA and ERK were detected in isolated mouse DRG neurons by immunostaining for phosphorylated TrkA (Y785/TrkB Y816) and phosphorylated 44/42 (Thr202/Tyr204), respectively. Neurons were detected by immunostaining for NeuN.

#### ERK activity and transcriptional activity.

ERK activity and stimulation of transcription were measured in cells expressing EKAR FRET biosensors or SRE-Luc2P biosensors, respectively.

#### Cell surface TrkA and NRP1.

Cells were transfected with SnapTag-TrkA and pcDNA3.1 or HaloTag-NRP1 and labeled using SNAP-Surface Alexa Fluor 488 and HaloTag Alexa Fluor 660. Fluorescence emissions were recorded.

#### Receptor-receptor BRET.

For end-point studies, HEK293T cells were transfected with a fixed concentration of NanoLuc-NRP1, NanoLuc-p75^NTR^, or NanoLuc-TrkA and increasing concentrations of SnapTag-TrkA or SnapTag-CALCRL. For kinetic studies, cells were transfected with NanoLuc-TrkA, SnapTag-TrkA, and pcDNA3.1 or HaloTag-NRP1. Cells were incubated with SNAP-Surface Alexa Fluor 488. Luminescence and fluorescence were measured.

#### BRET trafficking.

Cells were transfected with TrkA-HA-Rluc8 and fluorescent markers for the plasma membrane (RGFP-CAAX), early endosomes (tdRGFP-Rab5a), recycling endosomes (tdRGFP-Rab4a), or cis-Golgi apparatus (tdRGFP-Giantin). Cells were cotransfected with pcDNA3.1, HaloTag-NRP1, or siRNA. Cells were preincubated with inhibitors of endocytosis, GIPC1, and myosin VI or controls. Cells were incubated with coelenterazine and stimulated with NGF. Luminescence and fluorescence were measured.

#### qPCR.

Primers to human and mouse TrkA, NRP1, GIPC1, and GAPDH were used and the relative abundance of mRNA calculated (63).

#### Statistics.

Data are presented as mean ± SEM or ± SD. Differences were assessed using paired or unpaired 2-tailed *t* test for 2 comparisons, and 1- or 2-way ANOVA and Tukey’s, Dunnett’s or Šidák’s post-hoc test for multiple comparisons. *P* < 0.05 was considered significant at the 95% confidence level.

#### Study approval.

The University of Cincinnati Institutional Review Board deemed the collection of DRG from deidentified organ donors to be human subjects exempt (00003152, study ID 2015-5302). The New York University Institutional Animal Care and Use Committee approved mouse experiments (PROTO202000006).

#### Data availability.

Values for all data points in graphs are reported in the Supporting Data Values file.

## Author contributions

CJP, RT, ED, KG, ACR, RB, HB, LM, and AMM conducted experiments and analyzed results. HH and ARBT provided reagents. CJP, BLS, SD, ADG, RK, and NWB designed experiments, wrote the manuscript, and obtained funding.

## Supplementary Material

Supplemental data

Supporting data values

## Version 1 11/26/2024

In-Press Preview

## Version 2 02/17/2025

Electronic publication
